# Canine Leishmaniasis: An Overview of the Current Status and Strategies for Control

**DOI:** 10.1155/2018/3296893

**Published:** 2018-03-29

**Authors:** Raul Rio Ribeiro, Marilene Suzan Marques Michalick, Manoel Eduardo da Silva, Cristiano Cheim Peixoto dos Santos, Frédéric Jean Georges Frézard, Sydnei Magno da Silva

**Affiliations:** ^1^Departamento de Medicina Veterinária, Universidade Federal de Juiz de Fora, Campus Juiz de Fora, Rua José Lourenço Kelmer s/n, Campus Universitário, Bairro São Pedro, 36036-900 Juiz de Fora, MG, Brazil; ^2^Departamento de Parasitologia, Instituto de Ciências Biológicas, Universidade Federal de Minas Gerais, Belo Horizonte, MG, Brazil; ^3^Empresa de Pesquisa Agropecuária de Minas Gerais (EPAMIG), Campo Experimental de Pitangui, ITAC/EPAMIG, Pitangui, MG, Brazil; ^4^Departamento de Fisiologia e Biofísica, Instituto de Ciências Biológicas, Universidade Federal de Minas Gerais, Belo Horizonte, MG, Brazil; ^5^Departamento de Parasitologia, Universidade Federal de Uberlândia (UFU), Uberlândia, MG, Brazil; ^6^BrasiLeish Group, Study Group on Animal Leishmaniasis, Belo Horizonte, MG, Brazil

## Abstract

Canine leishmaniasis (CanL) is a vector-borne disease caused by* Leishmania infantum* and is transmitted by female phlebotomine sand flies primarily between animals and secondarily to humans. The course of infection may be different from one individual dog to another, ranging from spontaneous cure to acute evolution that leads to death, if proper management and therapy are not adopted. A parasitological cure is rarely achieved and clinical recurrences in CanL are frequent. Vaccination associated with the use of topical insecticides is undoubtedly the most effective form of prevention and control of the disease. In order to integrate the most important scientific knowledge of the literature in one objective publication, this review proposes a short overview of the main points of CanL.

## 1. Introduction

Leishmaniasis is a group of diseases produced by the invasion of protozoan parasites of the genus* Leishmania* into the mononuclear phagocyte system of mammalian hosts. They are transmitted primarily by the hematophagous activities of female phlebotomine sand flies belonging to the genera* Lutzomyia *(New World) and* Phlebotomus *(Old World). These neglected diseases are prevalent in at least 98 countries and 3 territories on 5 continents, of which the majority are underdeveloped countries [1, 2]. Approximately 12 million people are infected with a species of* Leishmania* at any given time [2].

About 70 species of mammals, including humans, are considered vertebrate hosts of different species of* Leishmania* around the world, and some of them are reservoirs of the parasite in nature [1]. Although the natural infection in rodents [3, 4] and canids [5–10] is more common, the parasite is able to infect xenarthrans [11, 12], hyraxes [13], marsupials [14], chiropterans [15–17], lagomorphs [18–21], procyonids [11, 22], felids [23–26], Perissodactyla [27, 28], and primates [11, 29]. Determining the precise role played by each host in the transmission cycle remains a challenge.

These protozoans cause a wide variety of clinical forms ranging in severity from self-healing cutaneous leishmaniasis (CL) to fatal disseminated visceral leishmaniasis (VL) [30]. Among the recognized clinical forms of the disease, kala-azar, or VL, is the most severe and progressive form, as it is almost always fatal if untreated. In the Indian subcontinent and East Africa, VL is transmitted between people (i.e., anthroponotic). In the rest of the world, particularly in the highlands of China, Central Asia, the Middle East, Transcaucasia, the Mediterranean, and Central and South America, VL is a zoonosis; that is, it is transmitted between animals and is secondarily transmitted to people [31].* Leishmania infantum* has been identified as the main aetiologic agent of canine leishmaniasis (CanL) [32], which is a major global zoonosis that is potentially fatal to humans and dogs [32], and it is one of the world's most important emerging diseases [1].

## 2. Transmission and Life Cycle

Since the discovery of CanL in Tunisia, by Nicolle and Comte (1908) [33], the dog has been implicated as a major reservoir of the etiological agent of VL, playing a key role in its transmission [34]. Other infected mammals, such as the crab-eating fox* Cerdocyon thous* and opossums* Didelphis* spp., are suspected as playing an epidemiologic role in transmission, but the confirmation of these hosts as reservoirs and their impact on the transmission cycle is unknown [7, 35, 36]. Maned wolves* (Chrysocyon brachyurus)* and bush dogs* (Speothos venaticus)* can be infectious to sand fly vectors even in the absence of clinical signs, but the epidemiological relevance of these findings has not yet been established [29, 37, 38]. The susceptibility of domestic cats* (Felis catus)* to infection by* L. infantum*, the clinical outcome, and their importance for the maintenance of the life cycle of the parasite are poorly understood [39]. It seems that the immune response in cats is effective enough to control the infection and confer a certain degree of resistance, if there are not immunosuppressive events such as retroviruses [Feline Immunodeficiency Virus (FIV) and Feline Leukemia Virus (FeLV)] [40], cancer, autoimmune disease, and others. Though infected domestic cats could be infectious to competent vectors of* L. infantum*, the confirmation of these hosts as accidental hosts and as secondary or alternative reservoirs requires further study [39].

Among the over 800 phlebotomine sand fly species estimated to exist, about 98 species are currently proven or suspected vectors of leishmaniasis [41]. Like many other vector-borne diseases, transmission originates during blood meals that females require to develop a batch of eggs. The parasite has a digenetic life cycle, alternating between a mammalian host and insect vectors. In short and according to the literature, when a sand fly bites an infected host, it also ingests macrophages infected by rounded and nonmotile amastigote forms. Then, the parasites transform from the amastigote to the flagellate promastigote stage, multiply by binary fission in the midgut, and migrate to the foregut and in mouth parts (pharynx, cibarium, and proboscis) of the infected sand fly vector. Subsequently, it can be transmitted to other new hosts, where these flies feed on blood meals, and the invertebrate cycle is concluded. When the infectious promastigote forms are inoculated from the vector's proboscis into the host's skin, they are phagocytized by macrophages. They then evolve into the amastigote form, where reproducing asexually and continuously in macrophages until rupture occurs. The parasites spread by invading mononuclear phagocytes in many organs, mostly spleen, liver, bone marrow, lymph node, and other tissues [7, 42–47].

Intriguingly, the occurrence of autochthonous cases of VL in places where the presence of phlebotomine has not been proven suggests other routes of transmission. Although non-sand fly transmission is reputed to be low, several studies have clearly shown the potential impact of nontraditional transmission routes in CanL, particularly sexual (venereal) and transplacental (vertical) transmission, which may have epidemiological significance in the dissemination and maintenance of disease, especially in the absence of the biological insect vector [48].

Sexual and transplacental transmission of* Leishmania* has already been reported in mice [49], humans [50–54], and dogs [55–59]. Genital lesions associated with VL have been well documented in dogs [60–62] and it seems that sexual transmission in dogs tends to be more efficient from the infected male to a susceptible female [63].* Leishmania* sp. was detected in many biological samples from stillborn or newborn puppies [64–66], symptomatic or asymptomatic naturally infected bitches [67], associated with necrotizing placentitis and abortion [68] or any gross or microscopic changes in the placenta [69]. Together these studies strongly support the notion that CanL is vertically transmitted. Other forms of transmission, such as infection during blood transfusion [70] or derivatives from infected donors [71, 72], organ transplantation [73, 74], and sharing of contaminated needles [75], should be carefully considered mostly in dog and human hosts. Additionally, a suspected mode of transmission is the direct dog-to-dog transmission of the parasite by wounds or dog bites [76, 77].

Other blood-feeding arthropods, such as ticks or fleas, have sometimes been suspected of transmitting* Leishmania* based on the association of CanL with the presence of these alternative vectors [78, 79]. Despite there being no definitive conclusion about the role of these ectoparasites in the transmission cycle of the disease [79, 80], it is nonetheless advisable to prevent and treat dogs against fleas, ticks, and mosquitoes [81].

## 3. Immunology and Clinical Signs

The number and intensity of clinical signs are determined by a set of factors involving parasite strain, genetics, and the host immune status. In this way, some dogs are able to control the infection for many years, without the appearance of clinical signs, and sometimes may even evolve spontaneous cure. On the other hand, some infected dogs may display an acute evolution and severe disease, or progressive course that leads inexorably to death, if proper management and therapy are not adopted.

The clinical diagnosis of CanL is complex, since almost 50% of the affected canine population does not exhibit clinical signs [92]. Moreover, when dogs are ill, they manifest a variable and nonspecific clinical spectrum [34], because CanL is a chronic and multisystemic disease that may potentially involve any organ [91].

**Figure 1:**
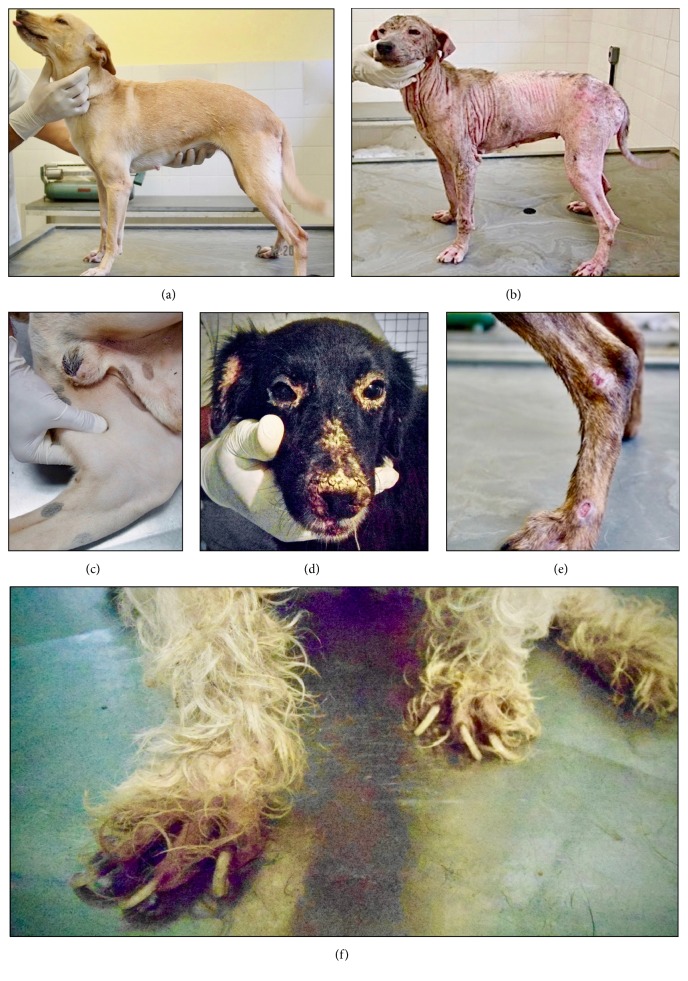
Clinical manifestations of dogs naturally infected with* Leishmania (Leishmania) infantum*: (a) asymptomatic dog (apparently healthy but infected); (b) generalized nonpruritic alopecia and multiple other dermatological abnormalities; (c) popliteal lymphadenomegaly; (d) bilateral blepharitis and extensive muzzle involvement with marked exfoliative ulcerative lesions; (e) ulcerative lesions at the bony prominences of the hind limb leg; (f) onychogryphosis. Photos by Raul Rio Ribeiro and Cristiano Cheim Peixoto dos Santos.

Clinical manifestations of dogs naturally infected with* L. infantum* are shown in Figure 1. Clinical signs may be present from three months to several years after dogs become infected [93]. In the classic cutaneovisceral form, one of the earliest and most common clinical signs of the disease is lymphadenopathy, mainly affecting the popliteal (Figure 1(c)), prescapular, and submaxillary lymph nodes [94]. Dermatological abnormalities occur later and are frequent and variable in their characterization and extension [95]. About 90% of these dogs present cutaneous lesions; however, dermatological alterations are rare in the absence of other signs of the disease [96]. The classic dermatological patterns include nonpruritic exfoliative dermatitis with or without alopecia, which can be localized or disseminated (Figures 1(b), 1(d)); erosive-ulcerative dermatitis (Figure 1(e)); nodular, papular, or pustular dermatitis; nasal hyperkeratosis (Figure 1(d)); nasal depigmentation and onychogryphosis (Figure 1(f)) [93, 97, 98]. Other signs involve anorexia, chronic enteritis and weight loss, splenomegaly and hepatomegaly, ophthalmopathy, and hypotrophy muscle [91, 93, 97, 99], as well as unusual or atypical signs like arthritis and neurological manifestations [100, 101].

Renal disease may be the sole clinical manifestation of CanL and it can progress from mild proteinuria to nephrotic syndrome or to an end stage renal disease [91]. Chronic renal failure is a severe result of disease progression and is the most common cause of death [91, 97].

There are two known clinical staging systems for CanL, with a good level of agreement between them [102], which contribute to the establishment of a more accurate diagnosis, prognosis, and treatment [103] by grouping the affected dogs according to the severity of their clinical presentation. In the LeishVet System, the disease is classified into four stages of evolution [Stage I: mild disease; Stage II: moderate disease (Substages A and B); Stage III: severe disease; Stage IV: very severe disease] based on physical examination and associated with the levels of antibodies determined by indirect immunofluorescence and biochemical-hematological findings [including detailed evaluation of renal function in conformity with International Renal Interest Society (IRIS)] [91]. The Canine Leishmaniasis Working Group (CLWG) System classifies dogs into five stages [Stage A: exposed dogs; Stage B: infected dogs; Stage C: sick dogs (dogs with clinically evident leishmaniasis); Stage D: severely sick dogs; Stage E: unresponsive to treatment or early relapse] according to clinical condition and associated with serological and parasitological (cytology, histology, or PCR) diagnosis and clinicopathological abnormalities [97, 104].

There remains no consensus on the exact relevance of each clinical form in the transmission cycle of the parasite. Some evidence suggests that the majority of transmission events to vectors result from a small proportion of infectious dogs with very high skin parasite loads, which would be correlated to severe disease [105, 106]. On the other hand, asymptomatic dogs could be also highly infectious, indicating their role in maintaining and spreading the parasite in endemic areas [107]. Despite these contradictory results and until specific and sensitive markers of infectiousness, whether direct or indirect, are available, it is prudent to consider that both symptomatic and asymptomatic dogs could be infectious to sand fly vectors and that they should therefore be considered equally when proposing control measures.

The immune mechanisms responsible for resistance or susceptibility to infection are not yet well known. The effectiveness of the immune response is a fundamental aspect in the pathogenesis of the disease and its progression [108], playing a crucial role in clinical manifestations of CanL.

In humans [109], mice [110], and dogs [93, 111] the protective immunity against leishmaniasis is mediated by T cells and is associated with the production of IFN-*γ* and TNF-*α*, while the role of Th2 cytokines, such as IL-4 and IL-10, and exuberant humoral response are related to progressive disease [93, 103, 111, 112].

It seems that the susceptibility to CanL of some breeds, such as Boxer, Cocker Spaniel, Rottweiler, and German Shepherd, can be associated with the expression of the Slc11a1 (Solute Carrier family 11a member 1; formerly NRAMP1) gene and/or major histocompatibility complex (MHC) class II polymorphism [113–116]. Conversely, the Ibizan Hound has been reported to be more resistant to* Leishmania *infection due to it displaying a predominantly cellular immune response [113, 117].

The greater rate of infection in working dog breeds is possibly due to more contact time with the insect vector in outside environments. Although controversial, the length of the coat can probably influence the risk of infection, since it is a characteristic that varies greatly among canine breeds. In short, it seems that the chances of acquiring* Leishmania* infection are lower in mixed-breed female dogs, with long hair, maintained in domestic-restricted or restrained (dogs raised indoors) without the presence of green surroundings close to home [118].

## 4. Laboratory Findings

The laboratory analysis of parameters related to hematopoiesis, renal function, and serum electrophoretic profile must be used in the clinical routine as a complementary tool in diagnosis. The marked polyclonal humoral response that occurs after infection gives rise to visible changes in the electrophoretic plasma profile and contributes to the occurrence of organs damage, such as kidneys, eyes, and skin. In addition, high parasite loads in the components of the mononuclear phagocyte system (MPS), for example, in bone marrow and liver, triggering the occurrence of clinical pathology related to hepatic and hematopoietic functions [34].

Anemia is one of the main laboratory findings on the hemogram. It is likely that more than one factor is involved in the etiology of anemia, such as hemorrhage, hemolysis, chronic renal failure, bone marrow hypoplasia, or aplasia, and decreased lipid fluidity of the erythrocyte membrane [34, 119, 120]. The fact that 50 to 70% of patients present normocytic/normochromic and nonregenerative anemia suggests, at the very least, the participation of chronic inflammatory disease and/or impairment of erythropoiesis due to infection-induced changes in bone marrow and/or kidneys [34]. Apparently, there is a relationship between anemia and clinical forms of the disease [34, 121, 122]. Bone marrow dysfunction does not usually involve precursor cells of leukocytes [34, 121], although dermatological lesions accompanied by secondary bacterial infections, or other comorbidities, can do so.

Dysproteinemia is considered one of the most important changes in the disease [34]. Protein imbalance is represented by the increase of total serum proteins (hyperproteinemia), hyperglobulinemia, and hypoalbuminemia, which also determines the inversion in the albumin/globulin ratio. Hyperglobulinemia is a result of the discrete or scarce increase of the *α* and *β* fractions accompanied by a significant increase of the *γ*-globulins, determining the hypergammaglobulinemia. The reduction of albumin levels is partly a result of renal excretion due to glomerular damage produced during the course of the disease and the low production by the liver in cases of liver failure.

CanL is often characterized by an increase in total serum proteins (hyperproteinemia), azotemia, hypergammaglobulinemia (polyclonal B cell response), hypoalbuminemia (renal and/or liver failure) [123], and values of A-G ratio below the lower limit of reference [34], since it is recognized that kidney damage associated with the disease is almost inevitable [34], which reinforces the fact that these parameters are good markers for diagnosis and therapeutic monitoring.

Renal disease in CanL may manifest as mild proteinuria to nephrotic syndrome or chronic renal failure, in which there is glomerulonephritis usually associated with the deposition of immune complexes in the kidneys. The activity of hepatic enzymes is generally within the reference values for the canine species, although biochemical findings in infected dogs can include alterations in aspartate aminotransferase, alanine aminotransferase, and alkaline phosphatase [123, 124].

## 5. Diagnosis

To improve the prognosis and to avoid both human and dog transmission (from false negative cases) and unnecessary euthanasia (from false-positive cases), diagnosis should be established as soon as possible, even on the basis of only a few or even a single clinical sign [42]. The diagnosis is made considering the epidemiological origin and the set of clinical signs presented by the dog [91]. Due to the large number of asymptomatic dogs and the absence of pathognomonic clinical signs, the diagnosis depends on laboratory support. All the parasitological, immunological, and molecular techniques available for diagnosis are important and need to be interpreted according to their benefits and limitations.

Parasitological diagnosis is the unique definitive method, which is often based on observations of amastigotes, preferentially in lymphoid organs such as bone marrow, lymph nodes, and spleen, as well as the liver and skin. In the clinical routine, a fragment obtained by skin biopsy allows the preparation of slides for cytological and histopathological/immunohistochemical techniques [125]. The aspiration biopsy from lymph nodes, bone marrow, or spleen can be evaluated by smears stained by Giemsa or Panoptic methods and, more rarely, in culture media (NNN, LIT, and *α*-MEM, among others). The sensitivity of the bone marrow smear is about 60–85% and 30–40% for lymph node [126]. According to the literature, splenic aspirates are considered as the method of choice for parasitological diagnosis in CanL [127].

Molecular techniques have high sensitivity and specificity, and PCR and qPCR are currently part of the veterinary diagnostic routine, which are especially useful for follow-up and may be performed on various biological samples, such as peripheral blood, bone marrow aspirate or lymph nodes, skin fragment, and others [91, 128, 129]. It is important to highlight that information provided by PCR/qPCR should not be separated from the data obtained from clinicopathological and serological evaluations [91].

CanL is frequently diagnosed through the detection of specific antibodies against* Leishmania* sp., preferably using quantitative serological techniques like immunofluorescence antibody test (IFAT) and enzyme-linked immunosorbent assay (ELISA). However, serological tests present important limitations, such as cross-reactions with* Trypanosoma* parasites, cutaneous leishmaniasis species, and other hemoparasites [130, 131], as well as false negative results in anergy cases or low titers (dubious reactions) [132].

Recently, immunochromatographic assays have been employed as routine laboratory tests in veterinary clinics for the detection of dozens of diseases including CanL. These tests are quick and easy (about 15 minutes) to perform, require no trained personnel or specialized laboratory training to interpret the results, and present reliable indexes of sensitivity and specificity. For CanL, usually recombinant proteins of the parasite, like rK39, are impregnated onto nitrocellulose membranes, and serum samples are applied in the rapid test platform. The Brazilian Ministry of Health officially established a rapid chromatographic immunoassay for canine survey based on dual path platform (DPP®) for disease screening and ELISA as a confirmatory test [133]. From the point of view of public health, positive results in serological tests are used as a criterion for indication of euthanasia in suspected dogs based on the elimination program for control of VL adopted in Brazil.

## 6. Treatment

Even though parasitological cures are rarely achieved, and clinical recurrences in CanL often occur after therapy, it is necessary to consider that the available protocols can promote clinical cure, increase the life expectancy, and improve the quality of life, in addition to reducing the parasite load and infectiousness to sand fly vectors. Thus, the decision to treat a diseased dog is the result of a discussion between the dog owner and the veterinarian. An important factor analyzed is the owner's ability and/or willingness to comply with the treatment protocol [42], in addition to the assessment of the dog's potential responsiveness to therapy by a complete serologic, hematologic, and biochemical profile and urine analysis in order to evaluate, principally, the bone marrow and renal and hepatic status. According to the literature, the clinical response to treatment can vary from poor to good depending on their overall initial clinicopathological status and their specific response to therapy. For instance, dogs with renal insufficiency are expected to have a lower recovery rate in comparison to those without compromised kidneys or only mild proteinuria [91]. For reasons of public health and to prevent reinfection, the constant use of permethrin spot-on and/or flumethrin or deltamethrin-impregnated collars in treated dogs and continuous veterinary monitoring is necessary.

Current treatment protocols are summarized in Table 1. Some chemotherapeutic compounds used in the treatment of CanL are included within the 19th edition of World Health Organization (WHO) Model List of Essential Medicines against leishmaniasis: pentavalent antimonials (Sb^v^), miltefosine, amphotericin B deoxycholate or formulated in liposomal formulations, and paromomycin [43]. In addition to the drugs mentioned, there are other products that are proposed to modulate the immune response, immunostimulating the animal organism, such as domperidone, cytokines, and vaccines (immunotherapy). However, in veterinary medicine, allopurinol (a purine analog) is considered the major first line drug for long-term treatment of CanL, often in combination with pentavalent antimonials or miltefosine for the first month and then continued alone [91, 134]. While it is rarely used for the treatment of human leishmaniasis, as allopurinol is the only drug recommended by the WHO for the treatment of CanL, recently the first report of resistance to allopurinol was published in* L. infantum* parasites isolated from dogs, and this was associated with clinical relapse [135].

Treatment of CanL with miltefosine (Milteforan®) was authorized in Brazil in 2017, a decade after its introduction in Southern Europe. However, after a six-year follow-up, clinical and laboratory findings indicated that meglumine antimoniate plus allopurinol had better clinical efficacy than miltefosine plus allopurinol in CanL [134].

The most frequently chosen treatment for CanL is antimoniate meglumine (pentavalent antimonial) administered subcutaneously at the dose of 100 mg/kg once a day for 1 month together with allopurinol (leishmaniostatical drug) administered orally 10 mg/kg every 12 hours for six months minimum [91, 134, 136] (Table 1). The duration of the treatment depends on the severity of the disease, individual tolerance of drugs, and clinical response to treatment. There are also several side effects, such as xanthinuria, renal mineralisation, and urolithiasis in the case of long-term treatment with allopurinol, and meglumine antimoniate can be potentially nephrotoxic and miltefosine can produce gastrointestinal upset [91, 129, 137].

Some immunomodulator-based treatments, like domperidone, can enhance innate defense mechanisms, activating phagocytic cells and potentiating the intracellular killing of the parasites, which can help to prevent CanL and reduce the risk of developing the clinical disease [138]. Recently, a study unprecedentedly registered the parasitological cure of dogs with VL treated with an innovative combined therapy with liposome-encapsulated meglumine antimoniate and allopurinol [139].

Knowledge about host–parasite relationships in dogs is increasing and signals the existence of factors inherent to the host, such as immunological differences in response to infection, which would influence the efficacy of the treatment. With this in mind, research groups seek the cure of dogs through new formulations of existing drugs or by associating them with immunostimulants and immunotherapeutics. The observed results indicate improved treatment in the future.

## 7. Prevention and Control

Considering that the sand fly bite is the most important route of transmission of CanL, the infection control measures should be primarily focused on preventing contact with the insect vector, either through physical barriers (fine mesh nets in windows and kennels), chemical barriers (repellents), or handling (avoiding exposure to twilight, eliminating organic peridomiciliary material). Predicting a large possibility of failure of these measures, the dog still needs to be able to respond to the infection challenge caused by the bites of infected sand flies, preferentially by an adaptive immune response previously developed through vaccination, or as a last alternative, by chemotherapeutics, which can boost the immune system to help fight infection.

Current prophylactic measures used for the prevention and control of CanL are summarized in Table 1. Repellent products available for preventing CanL contain synthetic pyrethroids (deltamethrin, permethrin, or flumethrin) alone or in combination with other insecticides, which displays a synergistic effect on insects. The protection effect against sand flies after use may range from 2–4 weeks in spot-on formulations to 4–8 months in impregnated PVC collars (Scalibor® and Seresto®), which must be used in both noninfected and infected dogs [140–143].

Vaccination against CanL is a recent tool for pet owners and unfortunately the two commercial vaccines available have low protective efficacy of about 68–71% (Canileish® 68.4%; Leish-Tec® 71%) [82, 84, 144].

There is no scientific evidence that seropositive dog culling could reduce the incidence of VL [145, 146], and wherever this has been applied (e.g., Brazil and Balkan and Central Asian countries), national programs for VL control have failed. Therefore, vaccination against* Leishmania* associated with topical insecticides is undoubtedly the most effective form of prevention and control of CanL.

## 8. Conclusions

CanL is a zoonotic chronic disease transmitted mostly by infected sand flies and can be potentially fatal to humans and dogs. Their epidemiological, clinical, and laboratory aspects are very variable, which makes it difficult for veterinary practitioners to complete a diagnosis and then treat and control the disease, especially due to the lack of more effective drugs and vaccines. However, considerable efforts are being made by professionals from multidisciplinary areas in order to improve the knowledge about this parasitic disease, so that prevention, treatment and control may be improved in the future.

## Figures and Tables

**Table 1:** Current treatment protocols and prophylactic measures used for the prevention and control of canine leishmaniasis.

Commercialized vaccines		
Trade name/licensed	Antigens/adjuvants	Efficacy in field studies [references]

CaniLeish®/Virbac	Excreted-secreted proteins of *L. infantum *(LiESP)*/*QA21	68.4% [82]
Leish-Tech®/Hertape Calier	Recombinant protein A2 of *L. donovani*/Saponin	71.4% [83]
Leishmune®/Zoetis (marketing temporarily suspended)	Fucose-Mannose Ligand (FML) of *L. donovani*/QS21	76–80% [84]
LetiFend®/Leti + MSD-Animal Health	Recombinant Protein Q from *L. infantum/*None	72%^*∗*^ [85]

Commercialized topical insecticides		

Trade name/licensed	Pharmaceutical compounds/application Form/duration	Efficacy in field studies [references]

Scalibor®/MSD-Animal Health	4% deltamethrin/impregnated PVC collar/4–6 months	50–86%; 61.8% [86, 87]
Seresto®/Bayer Animal Health	10% imidacloprid + 4.5% flumethrin/impregnated PVC collar/8 months	88.3% [86]
Advantix®/Bayer Animal Health	10% imidacloprid + 50% permethrin/spot-on/3 weeks	88.9–90.4% [88]
Exspot®/MSD-Animal Health	65% permethrin/spot-on/2-3 weeks	84% [89]
Frontect® or Frontline Tri-Act®/Merial	6.76% fipronil + 50.48% permethrin/spot-on/3 weeks	100% [90]
Effitix® or Fiprotix® or Fipratix®/Virbac	6.1% fipronil + 54.5% permethrin/spot-on/4 weeks	-
Perfikan®/Clément Thékan	6.1% fipronil + 54.5% permethrin/spot-on/4 weeks	-
Caniguard Line On®/Beaphar	40% permethrin/spot-on/5 weeks	-
Vectra 3D®/Ceva	4.95% dinotefuran + 36.08% permethrin + 0.44% pyriproxyfen/spot-on/4 weeks	-

Drugs and combinations used [43, 91]		

Active ingredient	Therapeutic protocols	Potential adverse effects

Allopurinol	10 mg/kg BID P.O. for at least 6–12 months or lifelong	Xanthine urolithiasis
Amphotericin B deoxycholate	0.5 mg/kg I.V. twice per week for 2 months	Nephrotoxicity
Meglumine antimoniate	75–100 mg/kg SID S.C. or 40–75 mg/kg SID S.C. for 4 weeks	Nephrotoxicity
Miltefosine	2 mg/kg SID P.O. for 28 days	Digestive disorders
Allopurinol + meglumine antimoniate	10 mg/kg BID P.O. for 12 months; 100 mg/kg SID S.C. for 4 weeks	Urolithiasis and nephrotoxicity
Allopurinol + miltefosine	10 mg/kg BID P.O. for 12 months; 2 mg/kg SID P.O. for 28 days	Urolithiasis and digestive disorders

^*∗*^Clinical effectiveness (prevention of clinical cases of leishmaniasis).
